# Effect of autophagy inhibition on chemotherapy-induced apoptosis in A549 lung cancer cells

**DOI:** 10.3892/ol.2013.1154

**Published:** 2013-01-25

**Authors:** FEIFEI LIU, DONGLEI LIU, YANG YANG, SONG ZHAO

**Affiliations:** Department of Thoracic Surgery, First Affiliated Hospital of Zhengzhou University, Zhengzhou 450052, P.R. China

**Keywords:** autophagy, chemotherapy, apoptosis

## Abstract

Chemotherapy is one of the main methods of cancer treatment and is known to induce autophagy in cancer cells. The main mechanism of chemotherapeutic agents is to promote apoptosis. In the process of chemotherapy, there is a unique association between autophagy and apoptosis. In this study, MDC staining, Hoechst 33342 staining and flow cytometry were used to explore the effects of autophagy on chemotherapy-induced apoptosis in A549 lung cancer cells and the association between autophagy and apoptosis was investigated via the addition of an autophagic inhibitor (3-methyladenine, 3-MA). This study demonstrated that cisplatin and paclitaxel were able to induce autophagy and apoptosis in A549 lung cancer cells and the inhibition of autophagy promoted cisplatin and paclitaxel-induced apoptosis. Furthermore, autophagy may play a protective role in the processes of cisplatin and paclitaxel-induced apoptosis.

## Introduction

Autophagy is a metabolic process in which autophagosomes combine with the lysosome in eukaryotic cells and degrade intracellular macromolecules and endogenous substrate to maintain a stable internal environment. During nutritional deficiency, autophagy provides nutrition (ATP, amino acids, etc.) for cell survival by degrading intracellular components (1). Autophagy is an important regulatory mechanism in cell growth, maturation and death and is associated with a variety of human diseases, including tumors.

Chemotherapy agents, including cisplatin and paclitaxel, may lead to an autophagic response, which is one possible method of inducing apoptosis, or may be associated with tumor resistance (2–5). Cisplatin and paclitaxel are commonly used in the treatment of lung cancer as first-line chemotherapeutic agents. Cisplatin induces apoptosis by interfering with DNA replication and also promotes autophagic cell death. Studies have shown that drugs, including cisplatin, are able to induce autophagy in cancer cells and autophagy may be associated with drug resistance in tumors (3–5). Paclitaxel is an effective mitotic inhibitor and apoptosis-inducing agent, which is used to treat malignant tumors and is widely used in lung cancer chemotherapy (6). Paclitaxel is able to maintain the stability of tubulin by promoting microtubule protein polymerization and inhibiting depolymerization. It is also known to induce apoptosis, thus it has become a first-line chemotherapeutic agent for non-small cell lung cancer. Furthermore, the effect of anti-cancer drugs on cancer cells may be increased by regulating the level of autophagy (7). It has been reported that paclitaxel induces autophagy and autophagic inhibition by small interfering RNA against the autophagic gene beclin 1, which may increase the rate of apoptosis induced by paclitaxel (6).

There is an urgent requirement to improve chemotherapy-induced apoptosis in cancer cells and increase the sensitivity of cancer cells to chemotherapeutic drugs in clinics. Therefore, we observed autophagy in A549 lung cancer cells, which was induced by chemotherapeutic drugs, either alone or in combination with an autophagic inhibitor (3-methyladenine, 3-MA), to provide a scientific basis for improving chemotherapeutic drug sensitivity.

## Materials and methods

### Cell and reagents

Human lung cancer A549 cells were obtained from The Cell Bank of Chinese Academy of Sciences (Shanghai, China). A549 cells were supplemented with 10% fetal bovine serum and antibiotics (100 U/ml penicillin and 100 *μ*g/ml streptomycin). Cells were incubated in a humidified incubator under 5% CO_2_ at 37°C. Cisplatin was purchased from Qilu Pharmaceutical Co., Ltd. (Shandong, China). Paclitaxel was purchased from Wanle Pharmaceutical Co., Ltd. (Shenzhen, China). 3-MA, Hoechst 33342, dimethyl sulfoxide (DMSO), monodansylcadaverine (MDC) and methyl thiazolyl tetrazolium (MTT) were obtained from Sigma-Aldrich (St. Louis, MO, USA).

### MTT assay for cell growth inhibition

Cells were seeded at a density of 1×10^5^ cells in each well of the 96-well plates and incubated for 24 h. A series of concentrations of cisplatin, paclitaxel or 3-MA were added to the wells for 24, 48 or 72 h. MTT (5 g/l, 20 *μ*l/well) was added to each well and incubated at 37°C for 4 h. DMSO was then added (100 *μ*l/well) to each well to dissolve any crystals and the plates were agitated for 10 min. Absorbance values at 490 nm were detected by the microplate reader. Cell growth inhibition was calculated on the basis of the following formula: Cell growth inhibition rate (%) = [1 − A490 (experimental group)/A490 (control group)] × 100. Each experiment was repeated three times.

### MTT assay for cell proliferation

The experiment was divided into five groups: the control group (without drug intervention), the 3-MA group (3-MA treatment alone), the cisplatin group (cisplatin treatment alone), the paclitaxel group (paclitaxel treatment alone), the 3-MA and cisplatin combined group (3-MA and cisplatin were added simultaneously) and the 3-MA and paclitaxel combined group (3-MA and paclitaxel were added simultaneously). Cells were plated as per the above assay. Following incubation for 24 h, the drugs were added according to the above experimental groups. Cells were incubated in a humidified 5% CO_2_ atmosphere at 37°C for 24 h. MTT and DMSO were added to the wells in succession. Cell proliferation was calculated using the following formula: Cell proliferation (%) = A490 (experimental group)/A490 (control group) × 100. Each group was assayed in triplicate.

### MDC staining

A549 cells in the logarithmic growth phase were treated with trypsin and plated in 24-well plates at a density of 1×10^5^ cells. Following incubation for 24 h, drugs were added at the corresponding concentrations to the five experimental groups. After 24 h, cells were stained with MDC (50 *μ*mol/l) for 60 min at 37°C, washed with PBS three times, fixed with 4% paraformaldehyde at 4°C for 15 min and washed with PBS a further three times. Inverted fluorescence microscopy was used to observe the change in autophagic vacuoles and to capture images. Cell fluorescence intensity in the various groups was measured by flow cytometry.

### Hoechst 33342 staining

Cells were plated in 24-well plates and incubated for 24 h. Drugs were added to each well according to the five experimental groups, and incubated for 24 h. The cells were washed with PBS three times and stained with Hoechst 33342 (1 mg/l) for 20 min at 37°C. Images of the Hoechst 33342 fluorescence were captured using inverted fluorescence microscopy, after the cells were washed with PBS three times. The fluorescence staining percentage of positive cells was calculated according to the images.

### Apoptosis detected by flow cytometry

Cells were seeded at 1×10^5^ in each well of the six-well plates and were pretreated with 3-MA, cisplatin or paclitaxel for 24 h, according to the five experimental groups. Cells were collected by trypsinization and washed with PBS. After staining with Annexin V-fluorescein isothiocyanate (FITC) and propidium iodide (PI) successively, the cells were immediately detected using flow cytometry.

### Statistical analysis

The results are expressed as the mean ± SD. SPSS 17.0 statistical software was used to analyze the results and a single-factor analysis of variance was used to compare the differences between groups. P<0.05 was considered to indicate a statistically significant difference.

## Results

### Cell growth inhibition and proliferation as detected by an MTT assay

**Figure 1:**
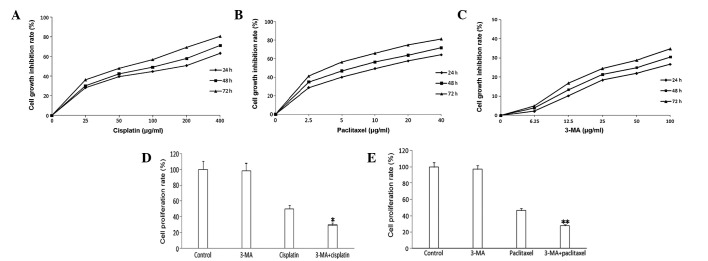
(A–C) Different concentrations of drugs and treatment times had varied effects on the cell growth inhibition rate. (D and E) Each group had a different impact on cell proliferation rate. ^*^P<0.05 compared with the cisplatin group; ^**^P<0.05 compared with the paclitaxel group. 3-MA, 3-methyladenine.

Different drug concentrations and treatment times have varied effects on cell growth. Results from the MTT assay showed that, as drug concentration and treatment time increased, the rate of cell growth inhibition also increased. The IC_10_ of A549 cells was 12.5 *μ*g/ml after treatment with 3-MA for 24 h. Following treatment with cisplatin or paclitaxel for 24 h, the IC_50_ of A549 cells were 200 and 10 *μ*g/ml, respectively. Therefore, the optimal concentrations of cisplatin, paclitaxel and 3-MA were 200, 10 and 12.5 *μ*g/ml, respectively (Fig. 1A–C). 3-MA had no significant effect on cell proliferation. Cisplatin or paclitaxel treatment lead to a marked decline in cell proliferation. Compared with cispatin or paclitaxel treatment, the rates of cell proliferation were markedly declined in the 3-MA combined groups (Figs. 1D and E).

### Chemotherapeutic drugs trigger autophagy in A549 cells

**Figure 2:**
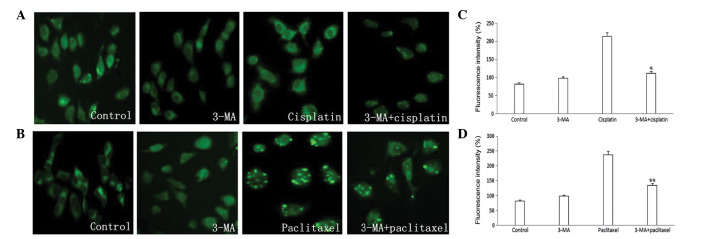
(A and B) Autophagy was detected by MDC staining. (C and D) Fluorescence intensity as detected by flow cytometry after treatment with different drugs in the absence or presence of 3-MA. ^*^P<0.05 compared with the cisplatin group; ^**^P<0.05 compared with the paclitaxel group. 3-MA, 3-methyladenine.

Due to the MDC gathered in autophagic vacuoles of the treated cells, blue-green and yellow-green punctate structures were visualized around the nucleus. No significant autophagic vacuoles appeared in the 3-MA group and compared with the control group, there was no significant difference in the MDC fluorescence intensity. Chromatin condensation and a large number of autophagic vacuoles appeared in the cisplatin and paclitaxel groups and fluorescence intensity was markedly increased. Compared with the cisplatin and paclitaxel groups, autophagic vacuoles were reduced and fluorescence intensity was decreased in the 3-MA combined group (Fig. 2).

### Apoptosis as detected by Hoechst 33342 staining in A549 cells

**Figure 3:**
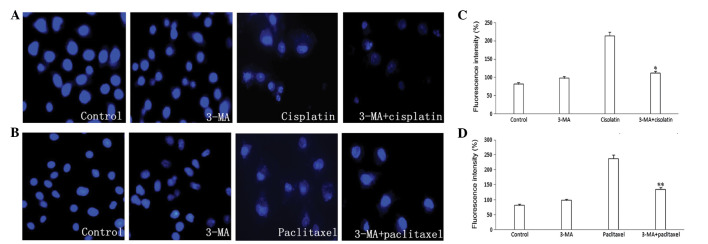
(A and B) Apoptosis induced by cisplatin or paclitaxel was detected by Hoechst 33342 staining in A549 cells. (C and D) The percentage of positive cells was calculated. ^*^P<0.05 compared with the cisplatin group. ^**^P<0.05 compared with the paclitaxel group. 3-MA, 3-methyladenine.

A change in cell morphology was not apparent in the 3-MA group. Results from Hoechst 33342 staining indicated that the percentage of positive cells was small. Nucleus cleavage and chromatin condensation appeared in the cisplatin and paclitaxel groups, which suggests that apoptosis occurred. In the 3-MA combined groups, apoptosis and the percentage of positive cells was increased significantly (Fig. 3).

### Inhibition of autophagy by 3-MA promotes chemotherapeutic drug-induced apoptotic cell death

Tumor cell apoptosis was detected using flow cytometry. Compared with control group, the 3-MA group showed no change in the apoptosis rate. The apoptosis rate in the combined 3-MA groups was significantly higher than in the cisplatin and paclitaxel groups. This indicates that 3-MA played a role in the process of promoting apoptosis.

## Discussion

Lung cancer is one of the most common malignancies worldwide. The failure of chemotherapy is often a result of drug resistance. Improving chemosensitivity is necessary for clinical improvements. Numerous studies have shown that autophagy, under specific treatment conditions, may be activated in various cancer cells and may play an important role in the process of tumorigenesis and tumor development (8). It has also been suggested that the development of malignant tumors is related to the disorder of autophagic regulatory mechanisms. However, the specific role of autophagy in the process of tumorigenesis and tumor development remains to be clarified.

Cisplatin is used for a variety of malignant tumors (including in lung cancer); it inhibits cancer cells during DNA replication and causes damage to the cell membrane structure, however, tumor resistance has been problematic in clinical treatment (9). Studies have shown that drugs, such as cisplatin, cause autophagy in tumor cells and that autophagy has a unique association with tumor resistance (3–5). Paclitaxel is able to maintain tubulin stability and inhibit cell mitosis, thus inducing apoptosis by promoting tubulin polymerization and inhibiting depolymerization. Adjusting autophagy appropriately may increase the cytotoxicity of anticancer drugs in tumor cells (7). In this study, we demonstrated that the autophagic inhibitor 3-MA significantly enhanced the cytotoxic effects of cisplatin and paclitaxel in A549 cells. This suggests that inhibitors of autophagy may be a novel sensitizer to improve the effects of chemotherapy drugs. Results from the MTT assays, Hoechst 33342 staining and flow cytometry show that autophagy, induced by cisplatin or paclitaxel, protected cancer cells from apoptosis and autophagic inhibitor 3-MA may potentiate the toxicity of cisplatin and paclitaxel and increase the rate of apoptosis. This indicates that autophagy is associated with chemoresistant mechanisms in A549 lung cancer cells.

During the course of chemotherapy, inhibition of autophagy to reduce the scavenging of damaged cells may allow an increase in the rate of apoptosis. Numerous studies have indicated that inhibition of autophagy increases the sensitivity of cancer cells to anticancer drugs, which causes DNA damage (10). This was consistent with previous studies, which suggested that the majority of chemotherapeutic drugs induce autophagy and inhibition of autophagy enhanced the effect of chemotherapy (11,12). In certain cases, autophagy and apoptosis have been induced at the same time (13,14) and autophagy has been shown to restrain apoptosis in specific circumstances (11,15,16).

In conclusion, our results indicate that autophagy was induced in lung cancer A549 cells, along with cisplatin- or paclitaxel-induced apoptosis and inhibition of autophagy was able to potentiate the toxicity of cisplatin and paclitaxel. They also demonstrated the existence of a connection between autophagy and apoptosis in certain cases. Thus we speculate that autophagy may play a protective role in the process of cisplatin- or paclitaxel-induced apoptosis, however, further study is required to determine whether autophagic inhibition could be utilized in clinics.

## Figures and Tables

**Figure 4:**
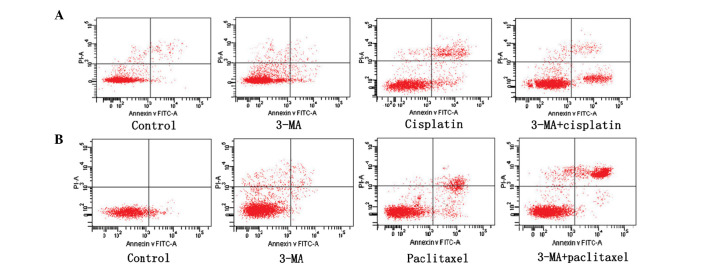
Cisplatin or paclitaxel was able to induce apoptosis in A549 cells and apoptosis was detected by flow cytometry. 3-MA, 3-methyladenine.
